# Polypharmacological Potential of Phosphodiesterase 5 Inhibitors for the Treatment of Neurocognitive Disorders

**DOI:** 10.14336/AD.2023.1129

**Published:** 2024-10-01

**Authors:** Ashish Kumar, Fred Kim, Dong-Keun Song, Jai Jun Choung

**Affiliations:** AriBio Co., Ltd., 56, Dongpangyo-ro, Bundang-gu, Seongnam-si, Gyeonggi-do, 13535, Republic of Korea

**Keywords:** Alzheimer’s disease, cGMP, mirodenafil, sildenafil, tadalafil, vardenafil

## Abstract

The prevalence of neurocognitive disorders (NCD) increases every year as the population continues to age, leading to significant global health concerns. Overcoming this challenge requires identifying biomarkers, risk factors, and effective therapeutic interventions that might provide meaningful clinical benefits. For Alzheimer’s disease (AD), one of the most studied NCD, approved drugs include acetylcholinesterase inhibitors (rivastigmine, donepezil, and galantamine), an NMDA receptor antagonist (memantine), and anti-amyloid monoclonal antibodies (aducanumab and lecanemab). These drugs offer limited relief, targeting singular pathological processes of the AD. Given the multifactorial nature of the NCDs, a poly-pharmacological strategy may lead to improved outcomes compared to the current standard of care. In this regard, phosphodiesterase 5 (PDE5) inhibitors emerged as promising drug candidates for the treatment of neurocognitive disorders. These inhibitors increase cGMP levels and CREB signaling, thus enhancing learning, memory and neuroprotection, while reducing Aβ deposition, tau phosphorylation, oxidative stress, and neuroinflammation. In the present article, we evaluate the therapeutic potential of different PDE5 inhibitors to outline their multifaceted impact in the NCDs.

Neurocognition refers to brain functions involving the speed of information processing, vigilance/attention, verbal learning/memory, working memory, visual learning/memory, reasoning, problem-solving, and verbal comprehension. These functions are integral to the brain and alterations lead to neurocognitive disorders (NCDs) [1]. Previous studies from animal models and clinical cases showed deposition and poor clearance of various toxic proteins like amyloid-β (Aβ), phosphorylated tau, and α-synuclein, cause mitochondrial dysfunction, proteasomal abnormalities, autophagy-lysosome system malfunction, neuroinflammation and neuron death as other common pathological features in NCDs [2]. Further, significant vascular dysfunctions, including blood-brain barrier (BBB) and cerebral blood flow impairment, are also associated with NCDs [3].

NCDs are progressive in nature and require urgent attention due to their deleterious effects and socio-economic burden on caregivers and healthcare services throughout the globe. Current FDA-approved drugs for AD treatment, e.g., acetylcholine inhibitors (donepezil, galantamine and rivastigmine), NMDA antagonist (memantine), and monoclonal antibodies (aducanumab and lecanemab) provide only a limited relief to the suffering population with substantial side effects. Further, complex and polygenic nature of these disorders led to high failure rates of drugs in various stages of clinical trials making it the most challenging field of the drug development. Multi-mechanism therapeutic approaches, in particular, the compounds with poly-pharmacological properties might have effective and promising future as drug candidates for such multifaceted disorders.

In this regard, phosphodiesterase type 5 (PDE5) inhibitors, which are used for the treatment of erectile dysfunction (ED), gained attention as potential therapeutic agents for NCDs. These drugs enhance the NO/cGMP/PKG/CREB signaling pathway by increasing cGMP levels [4]. Pharmacological analysis revealed low IC_50_, rapid absorption, high brain penetration, good tolerability, safety and efficacy of PDE5 inhibitors [5-7]. Here, we have included the effects of sildenafil, tadalafil, vardenafil, avanafil, yonkenafil and mirodenafil treatment for cognitive enhancement and amelioration of cognitive deficits in preclinical and clinical studies.

## Effect of PDE5 inhibitors on normal cognitive functions

Initial studies on PDE5 inhibitors on cognitive performance of healthy subjects showed that treatment of sildenafil enhanced ability to focus attention on a spatial auditory attention and a visual word recognition task in healthy young males [8]. In animals, sildenafil and vardenafil treatment improved reference memory [9-11], spatial memory [11-13] of rodents as well as cynomolgus macaques [14], increased long-term potentiation (LTP) and glutamatergic α-amino-3-hydroxy-5-methyl-4-isoxazolepropionic acid receptor (AMPAR) level [11, 13]. These studies have clearly demonstrated that PDE5 inhibitors have potential to enhance cognitive performance of healthy subjects.

### Effect of PDE5 inhibitors in the NCDs-related preclinical models

Preclinical studies of PDE5 inhibitors in cellular and animal models including AD, senescence, ischemic stroke [15], chronic cerebral hypoperfusion [16], and Huntington’s disease (HD) [17], etc. showed promising polypharmacological effects on neuroprotection, anti-inflammation, abnormal protein (Aβ and hyper-phosphorylated tau) depositions, regulation of ion channels, cerebrovascular maintenance, and cognitive functions [4, 18-21].

Cell culture studies have shown that sildenafil attenuated the advanced glycation end-products (AGE)-induced alterations in MTT level, intracellular ATP concentration, ROS formation, mitochondrial permeability transition, cytochrome C release and activation of caspase-3 in HT-22 cells [22, 23]. In AD patient-derived iPS cells, sildenafil treatment showed reduction in hyperphosphorylated tau levels and enhanced neurite growth in the neurons [24]. Mirodenafil treatment significantly enhanced cGMP levels, and ameliorated CREB/BDNF signaling (CREB phosphorylation, NGF and BDNF expression), abnormal protein aggregation (Aβ and hyperphosphorylated tau), mitochondrial membrane potential, apoptosis (caspase-3 and PARP levels), autophagy (LC3B)-II and p62 expression), phosphorylation of kinases [GSK-3β, Akt and AMPK], and expression of AD-associated genes including APP and BACE1 in neuronal cells treated with Aβ_1-42_ [18].

In animal models, most of the studies have shown that PDE5 inhibitors ameliorated abnormal proteins aggregation, apoptosis, autophagy, neuroinflammation, oxidative stress, and enhanced neurogenesis, CREB/BDNF signaling, and kinase pathways which are modulated in NCDs. For abnormal aggregation of proteins, the major hallmarks of NCDs, treatment of sildenafil [25-29], tadalafil [26, 30, 31], avanafil [32], yonkenafil [33], or mirodenafil [18] showed reduction in Aβ and hyperphosphorylated tau proteins levels. Treatments of sildenafil [15, 25, 34,], tadalafil [31], avanafil [32] or yonkenafil [33] also reduced neuroinflammation by decreasing astrocytes and microglia over-activation, and pro-inflammatory markers levels (IL-1β, IL-6, and TNF-α levels). Lipid peroxidation (MDA) and nitrite levels, the important cellular parameters for oxidative stress, were reduced by the treatment of PDE5 inhibitor tadalafil [35]. Sildenafil [27], or yonkenafil [33], treatments showed reduction of apoptosis in the animal models of AD.

Further, treatment of sildenafil [17], tadalafil [30, 31], zaprinast and UK-343664 [36], or yonkenafil [37] showed that PDE5 inhibitors increase neurogenesis and/or synaptic functions in the animal models of NCDs. Analysis of CREB/BDNF signaling demonstrated that treatments of sildenafil [27, 38, 39], or tadalafil [31] ameliorated CREB phosphorylation, Arc, and BDNF levels in AD-related rodent models. Amelioration of autophagy-related proteins level was demonstrated by tadalafil [31] treatment. Modulation of kinases (phosphorylation of Akt and GSK3β levels) was also reported to be restored by the treatment of sildenafil [25, 28, 29, 34, 40], tadalafil [31], and yonkenafil [33]. Sildenafil treatment also ameliorated APP and BACE1 expression [28, 29]. Vardenafil treatment showed amelioration of reduced proteasome activity in aluminium chloride/D-galactose-induced rat model of AD [41]. Treatment of sildenafil also showed increased angiogenesis and cerebral blood flow in the animal models of ischemic stroke [15] and HD [17].

**Figure 1.**
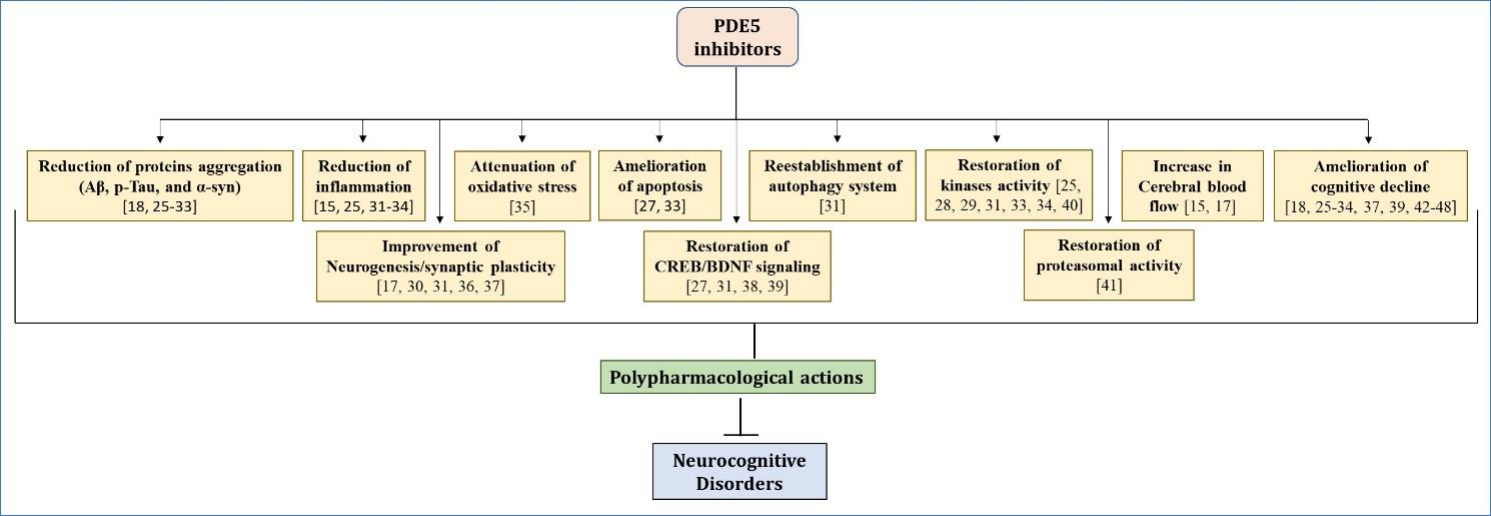
Polypharmacological action of phosphodiesterase 5 inhibitors for the treatment of neurocognitive disorders

Behavioral analysis has confirmed that the PDE5 inhibitors also have the potential to rescue the cognitive deficits in a wide variety of rodent models of cognitive deficit. Sildenafil treatment showed amelioration of memory impairments in various rodent models including AD transgenics [25, 26, 37], HD model (R6/1 mice) [42], senescence models [27-29,], bilateral ovariectomized female rats [39], diabetic and electroconvulsive shock-induced rat model [43], hyperammonemia and portacaval anastomosis rat models of hepatic encephalopathy [44], N(omega)-nitro-L-arginine methyl ester (L-NAME)-induced model [45, 46], and the cognitively impaired progenies of pre-eclampsia mothers [47]. Similarly, tadalafil treatment showed short-term and spatial memory improvement in rodent models like AD transgenic mice [26, 30], streptozotocin-induced model [31], senescence mice [34], and hepatic encephalopathy models [48]. Yonkenafil treatment in streptozotocin injection (i.c.v.) rat model of AD [33, 37] and mirodenafil administration in APP-C105 Tg AD mice [18] also showed amelioration of cognitive deficits. The ameliorative effects of PDE5 inhibitors are summarized in Figure 1.

### Effect of PDE5 inhibitors in the NCDs-related clinical studies

While PDE5 inhibitors have been studied in several diseases, their utility in neurocognitive disorders has only recently begun to be investigated. PDE5 inhibitors showed high clinical efficacy (~70% versus ~ 35% in placebo) with well tolerability and no serious side effects in the earlier clinical studies for ED [49]. Some side effects like flushing, headache, dyspepsia, nasal congestion, and nasopharyngitis have been reported in patients. Rare side effects like sudden loss of vision and non-arteritic anterior ischemic optic neuropathy, may happen in patients with hyperlipidemia, hypertension, and diabetes, were reported for sildenafil and tadalafil, and myalgia and back pain with tadalafil treatment in some cases. Some rare serious hypersensitive conditions like exfoliative dermatitis and Stevens-Johnson syndrome have been associated with sildenafil and tadalafil treatment [50-55].

Clinical studies of sildenafil (50 mg, one-time oral dose) treatment in AD patients showed amelioration of hippocampal fractional amplitude of low-frequency fluctuations (fALFF), cerebral blood flow, cerebrovascular reactivity, and cerebral metabolic oxygen rate [56-58]. For tadalafil, clinical studies showed significant improvement in the saturation level of blood oxygen and cerebral blood flow by tadalafil treatment (20 mg, one-time) in progressive cerebral small-vessel disease [59, 60], and improved cerebral blood flow in the brain as well as cognitive performance with tadalafil treatment (5 mg, once a day for 8 weeks) in ED patients with mild cognitive impairment [61]. However, vardenafil treatment showed no effect on memory performance in occasional cannabis users as well as healthy adults [62, 63].

Fang et al. (2021) conducted a pharmaco-epidemiologic analysis by endophenotype disease module. In this study, usage of sildenafil showed decreased risk of AD (~69%), suggesting it could be a potential modifier of disease risk [24]. Conversely, Desai et al. (2022) reported in a meta-analysis of patients with AD and pulmonary arterial hypertension that sildenafil or tadalafil treatment have no significant protective effects on reducing the risk of AD or other NCDs [64]. Also, they failed to find any ameliorating effects of sildenafil on exogenous Aβ_1-42_ levels in SH-SY5Y, BV2 microglial, or APP overexpressing H4-hAPP neuroglioma cells [24]. Desai et al. (2022) compared incidence of Alzheimer’s disease and related dementia (ADRD) after PDE5 inhibitor initiation versus endothelin receptor (ET) antagonist initiation among patients with pulmonary hypertension [64]. It has been reported that PDE5 inhibitors and ET antagonists [65], both can increase cerebral blood flow (CBF) and improve cognitive functions in AD animal models. Thus, it would be difficult to evaluate the difference in efficacies between PDE5 inhibitors and ET antagonists in terms of ADRD progression.

In a phase 2 clinical study (NCT03625622) in participants with mild to moderate AD, mirodenafil treatment improved ADAS-Cog13 score in mild AD subjects over 52 weeks and demonstrated robust safety and tolerability profile [www.alzforum.org/therapeutics/ar1001]. Mirodenafil also significantly reduced plasma pTau-181 for both 10 and 30 mg arms at week 52. Currently, mirodenafil is being studied in a global phase 3 trial (NCT05531526) for early AD.

Preclinical and clinical studies have shown positive effects of PDE5 inhibitors in AD. However, the complex nature of NCDs needs detailed analysis on the mechanistic pathways and off target effects of PDE5 inhibitors. Moreover, the clinical trials are limited to small cohorts, and are shown to be more effective in mild AD subjects. Therefore, more clinical studies are warranted to understand the effects of PDE5 inhibitors in large cohort size including moderate and severe subjects of AD as well as other NCDs.

### Conclusion

Here, we have systematically examined the emerging role of PDE5 inhibitors, specifically sildenafil, tadalafil, vardenafil, and mirodenafil, in the context of neurocognitive disorders. Both preclinical and clinical studies highlight the substantial potential of these agents in modulating key pathological features of NCDs, such as oxidative stress, mitochondrial dysfunctions, the accumulation of Aβ42 plaques and neurofibrillary tangles of pTau, neuroinflammation, neurodegeneration, synaptic loss, decreased cerebral blood flow, and memory impairment. The consistent safety profile of PDE5 inhibitors in various clinical studies combined with their multifaceted therapeutic impact, positions PDE5 inhibitors as promising poly-pharmacological agents for the treatment of NCDs. However, the complexity of NCD pathologies necessitates a deeper understanding of the mechanistic pathways through which PDE5 inhibitors exert their effects. Therefore, additional studies are required to further elucidate the role of PDE5 inhibition in NCDs.
